# Active Patient Participation in the Development of an Online Intervention

**DOI:** 10.2196/resprot.3695

**Published:** 2014-11-06

**Authors:** Inge Renske van Bruinessen, Evelyn M van Weel-Baumgarten, Harm Wouter Snippe, Hans Gouw, Josée M Zijlstra, Sandra van Dulmen

**Affiliations:** ^1^NIVEL (Netherlands Institute for Health Services Research)UtrechtNetherlands; ^2^Radboud University Medical CenterDepartment of Primary and Community CareNijmegenNetherlands; ^3^HematonAmersfoortNetherlands; ^4^VU University Medical CenterDepartment of HematologyAmsterdamNetherlands; ^5^Buskerud and Vestfold University CollegeDepartment of Health ScienceDrammenNorway

**Keywords:** communication, malignant lymphoma, online intervention, self-help application, patient participation, intervention development

## Abstract

**Background:**

An important and challenging part of living with cancer relates to the repeated visits to the hospital. Since how patients cope between these post-diagnostic visits depends partly on the information and support received from their physician during the visits, it is important to make the most of them. Recent findings reinforce the importance of training not only the health care professionals in communication skills, but providing patients with support in communication as well. Delivering such supportive interventions online can have potential benefits in terms of accessibility, cost-effectiveness, and ability to tailor information to personal needs. However, problems with attrition (dropout, non-usage) during the test phase and poor uptake after implementation are frequently reported. The marginal level of engagement of the patient as end user seems to play a role in this. Therefore, recent research suggests integrating theory-based development methods with methods that promote involvement of the patient at an early stage. This paper describes a participatory protocol, used to let patients guide a theory-informed development process.

**Objective:**

The objective of this project was to apply a bottom-up inspired procedure to develop a patient-centered intervention with corresponding evaluation and implementation plan.

**Methods:**

The applied development protocol was based on the intervention mapping framework, combined with patient participatory methods that were inspired by the participation ladder and user-centred design methods.

**Results:**

The applied protocol led to a self-directed online communication intervention aimed at helping patients gain control during their communications with health care professionals. It also led to an evaluation plan and an implementation plan. The protocol enabled the continuous involvement of patient research partners and the partial involvement of patient service users, which led to valuable insights and improvements.

**Conclusions:**

The applied protocol realized patient participation on different levels throughout the entire project. Early involvement, involvement on different levels, and flexibility in terms of planning and setup seem to be preconditions to creating a bottom-up inspired development procedure with (seriously ill) patients. Further research is necessary to find out if a more patient-centered approach improves the implementation and uptake of eHealth interventions.

**Trial Registration:**

Netherlands National Trial Register ID number: NTR3779; http://www.trialregister.nl/trialreg/admin/rctview.asp?TC=3779 (Archived by WebCite at http://www.webcitation.org/6TdfALKxV).

## Introduction

An important and challenging part of living with cancer concerns the repeated visits to the hospital. These visits are important as they monitor the development of the disease and set the stage for how to cope with life until the next consultation. Since how patients cope between these post-diagnostic visits depends partly on the information and support received from the health care professionals (HCPs) (eg, specialists, nurses) during the visits, it is important to get the most out of them. Many training programs are designed to improve HCPs’ communication skills, which may facilitate patient engagement in the medical dialogue. However, cancer patients ascribe many barriers in medical communication to their own attributes, such as a lack of communication skills, and interfering emotions and beliefs [1,2]. These findings reinforce the importance of training not only HCPs in communication skills, but providing patients with support in communication as well. Epstein and Street (2007) have stressed the need for developing specific types of interventions to support cancer patients, such as in-person coaching, interactive computer programs, videos of role models, and question prompt sheets [3].

Such interventions can be especially efficient when delivered online. The content and type of online interventions can be computer tailored to patients’ preferences and needs and they can be accessible any time and any place in a cost-effective way [4]. With regard to knowledge and skill building, the effects of online interventions for patients seem to be equivalent to traditional medical education methods (eg, a brochure or human-delivered intervention) [5,6]. Despite these potential benefits, problems with attrition (dropout, non-usage) during the test phase and poor uptake after implementation are frequently reported [7-9]. According to Eysenbach, characteristics related to the participants, the intervention, and the study design influence the usage and adoption success of online interventions [10].

The technology- and expert-driven development methods (top-down) are indicated as possible causes for attrition and adoption problems [11]. These imply a marginal level of engagement of the involved end-users (especially patients). Therefore, recent research suggests integrating methods that promote involvement of the patient at an early stage (bottom-up) with theory-based intervention development methods [11,12]. Patient participation is frequently referred to, the potential benefits are widely accepted, and there is a clear urge for more patient involvement [13]. However, the actual operationalization, that is, how and when (seriously ill) patients are involved, is rarely reported [14,15]. It often seems a more symbolic statement or it is used to describe the participation of patients in health programs. This differs from patients’ active involvement in the organization, goal setting, planning, and execution of interventions [16].

Considering the fact that the contribution of patients in oncology consultations is often limited [3,17,18] and that patients ascribe many communication barriers to personal attributes [1,2], the PatientTIME project was set up (Patients Talk In Medical Encounters). In this project, an online intervention is developed, tested, and implemented that aims to teach patients to take more control during their consultations. The project aims to realize this with a bottom-up inspired approach, which implies the involvement of seriously ill patients throughout the entire project. The initiation of the project was triggered by a specific request for support in communication with HCPs, expressed by a group of patients diagnosed with malignant lymphoma. Lymphoma patients often face long, intense treatment periods and/or monitoring periods under specialist care, which involve many hospital visits. Apparently, despite the (mainly paper-based) information available for this group, patients with malignant lymphoma experience difficulties in communicating their own agenda and needs to their HCP.

This paper outlines the patient participatory approach used to develop an online intervention with corresponding evaluation and implementation plan. The goal of this paper is to share the applied protocol, the use of the protocol in the PatientTIME project, and our lessons learned in the attempt to create a bottom-up inspired intervention.

## Methods

### Outline

**Figure 1:**
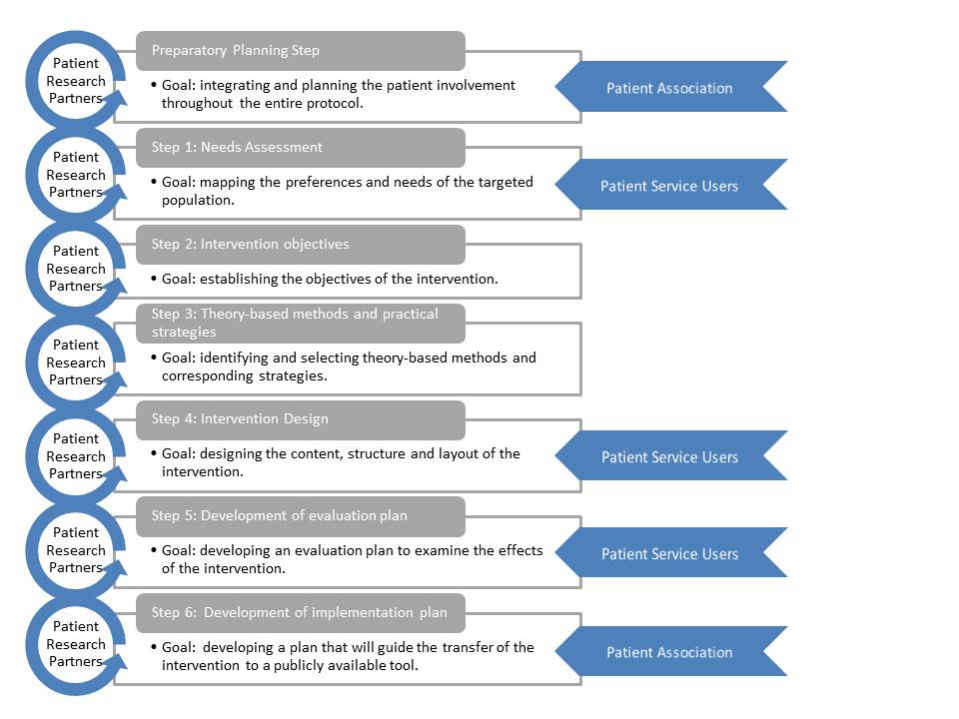
Stepwise protocol.

A stepwise protocol (Figure 1) was applied to develop the intervention with corresponding evaluation and implementation plan. For each step, goals were set and the procedure to involve patients was planned in advance. The Intervention Mapping (IM) framework was used as theoretical backbone of the protocol. Aiming at a patient-driven development protocol, practical patient participatory methods were integrated in the theoretical IM framework and used to inspire when and how patients could be involved.

### Intervention Mapping as a Theory-Based Guideline

**Figure 2:**
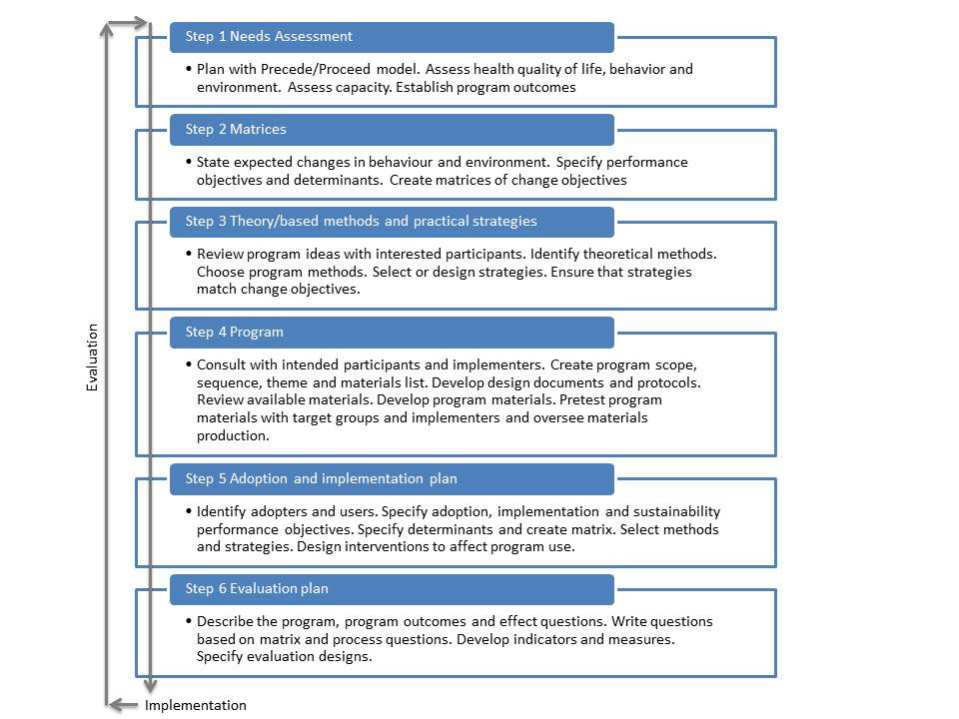
Intervention Mapping framework.

The IM framework systematically guides the planning and decision-making process in health promoting programs [19]. It comprises six steps in the process toward the development of a theory-driven and evidence-based intervention (Figure 2). The outcome of each step guides the next step. The IM framework has already been used successfully in developing a range of eHealth programs [20-23]. The IM framework was chosen as a guideline because it links decisions, final materials, and activities to theory. A preparatory step was added to the IM framework to plan and prepare the patient participation throughout the entire protocol.

### Patient Participatory Methods

The way patients were involved in the applied protocol was inspired by the concept of participation ladders. Different participation ladders describe the idea of involving participants in varying degrees [24-27]. Definitions of these degrees vary, but they all describe a stepwise scheme from no participation (eg, patients participate but have no understanding of the project; they get information but there is no dialogue) to the highest possible level of participation (participants directly collaborate with the stakeholders; have an agenda-setting, initiating role). This concept inspired us to involve patients on different levels and we operationalized this by (1) setting up a close collaboration with the patient association for malignant lymphoma (Hematon), (2) recruiting patients as research partners, and (3) planning the involvement of patient service users. Hematon informs and supports patients and champions patient interests. Patient research partners are involved throughout an entire project and they are equal partners in a working group. Patient service users are involved on different levels, in different parts of the project.

User-centered design (UCD) was used as a guide to realize patient participation in the different protocol steps. UCD is defined by Preece et al (2002) as “an approach, which views knowledge about users and their involvement in the design process as a central concern”. The challenge of UCD is to map the needs, behavior, actions, and abilities of the end user and let this information influence how the intervention takes shape. The context mapping method (Step 1) and the usability tests (Step 4) were inspired by UCD thinking.

### Patient Recruitment

All participating patients were adults diagnosed with malignant lymphoma and they all voluntarily signed up to contribute to the project. They were recruited via social media, online newsletters, advertisements on Hematon’s website, regional and national patient conferences, and leaflets in hospital waiting rooms. To recruit patient research partners, Hematon informed several of their active volunteers (patients) who had experience in information and communication technology (ICT) development and with supporting fellow patients.

### Project Management

A multidisciplinary working group consisting of researchers, HCPs, and a patient research partner was responsible for the daily coordination of the project. The working group collaborated with physicians, nurse practitioners, patients, user-interaction designers, software developers, and representatives of Hematon. Final decisions regarding the protocol were reached through discussions in the working group. Decisions related to the implementation plan were made in consultation with Hematon.

## Results

### Overview

The intervention development protocol resulted in three products: a self-directed online communication tool, a corresponding evaluation plan, and an implementation plan. The goal of the intervention is to help patients gain more control in the communications with their HCPs. Patients can access the intervention before each hospital visit. The information is provided via an algorithm computer-tailored to the patient’s self-assessed, momentary efficacy for communication with their HCP, to whether he or she attends the HCP alone or with a companion, and to the stage of treatment. The central information consists of short video clips of simulated consultations that model adequate communication behavior. Additionally, the intervention includes an open question prompt sheet (QPS), a reminder system linked to a list of planned hospital visit dates, and an option to store and play back audio recordings of the consultation (see Multimedia Appendix 1). The evaluation plan comprises a randomized controlled trial (RCT) protocol, in which the effects of the intervention on the patients’ perceived efficacy are measured in a trial setting. In the implementation plan, the conditions are built to transfer the evaluated intervention to a publicly available tool. The following paragraphs outline how the patient participatory protocol was used to develop these three products.

### Patient Participation Planning (Preparatory Step)

The goal of this preparatory step was to integrate and plan the patient involvement throughout the entire protocol. This resulted in the recruitment of two patients as research partners. They both had been active in supporting fellow patients and therefore they had built a rich body of knowledge about the different aspects of having malignant lymphoma. Additionally they both had a relevant professional background in ICT (Web-development, system design, research and development). One research partner (HG) became part of the working group. The second research partner was consulted on a more irregular basis. The research partners were directly involved in the planning of the PatientTIME project and in the decision-making processes in each protocol step. This involvement approach aligns with the upper steps of the participation ladder as they had an initiating and agenda-setting role and they worked directly with the other stakeholders. Additionally, patient service users were invited to participate in the needs assessment (Step 1), intervention design (Step 4), and the evaluation (Step 5). Moreover, their input was used to inspire the other protocol steps. Last, representatives of Hematon were consulted to explore the possibilities for implementing the intervention after the research project has ended (Step 6) and how we could use their network to keep in close contact with patients.

### Needs Assessment (Step 1)

The goal of the needs assessment step was to map the patient-perceived barriers and facilitators in communication with HCPs and to learn from patients’ experiences. A qualitative two-step method was applied, inspired by user-centered design thinking. The applied method is derived from the context mapping framework, used by product developers and user interaction researchers to gain insight into the needs of prospective users of new products [28]. Details of this study are described elsewhere [2]. In short, patients completed a set of assignments about their experiences with medical consultations, aiming to trigger them to verbalize and reflect on experiences, preferences, and needs without the presence of researchers or other patients. This so-called sensitizing process is supposed to enhance the quality and quantity of patients’ contributions in later (group) interviews [29]. Subsequently, these patients and their spouses shared their experiences during semi-structured (group) interviews, which were audio-recorded. Before conducting this needs assessment, a patient research partner reflected on the study design and the formulated questions. According to his feedback, the introduction was changed to further clarify the goals of the study, more and other examples were added to illustrate the questions, and subtle changes were made to the formulation of questions (eg, avoiding medical jargon, less formal style). A total of 37 patient service users (28 patients and 9 spouses) contributed to this needs assessment. They were open, willing, and motivated to share their experiences and they all had experienced difficulties in communication during consultations. Many communication barriers were ascribed to their own attributes (eg, emotions, skills, and beliefs).

**Figure 3:**
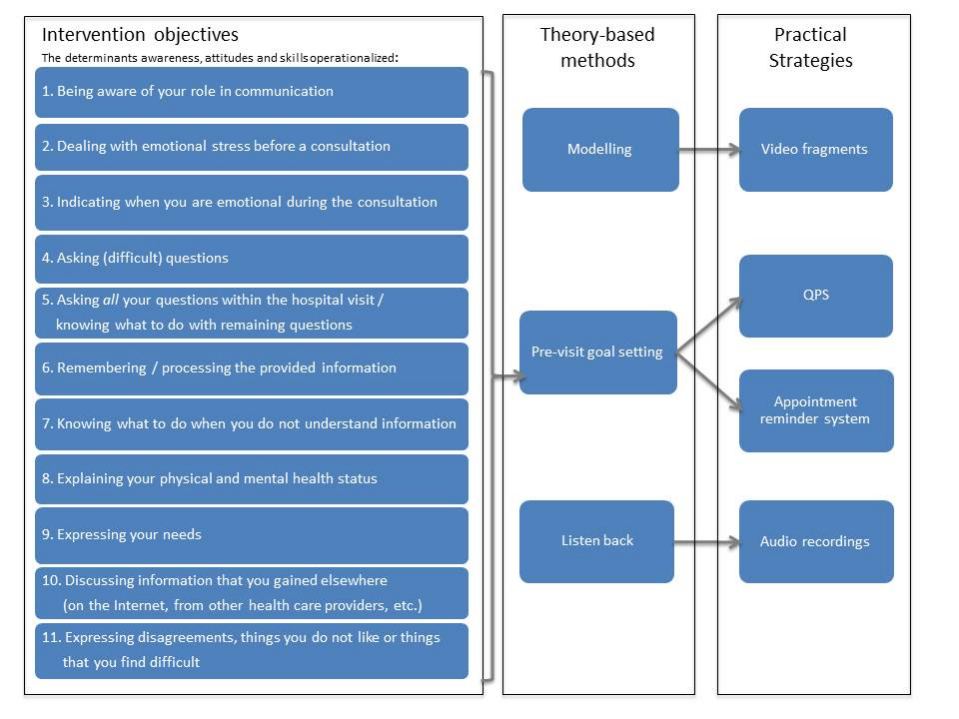
Intervention objectives, theory-based methods, and practical strategies.

The expressed barriers were analyzed, clustered, and translated into a list of intervention objectives (Step 2) and used as a basis for the central information of the intervention (Step 4). For example, patients did not want to be bothersome and therefore they found it hard to ask (all of) their questions and to express details about their physical and/or mental health status. This information was used to develop information about how to request attention for your prepared questions (Figure 3, Objective 4) and about the importance of expressing your physical complaints and worries (Figure 3, Objective 8). Participants also reported that their communication attitude and skills changed over time, and so did their perceived barriers and facilitators. This finding stressed the need to inquire about the patient’s needs before every hospital visit and tailor information accordingly.

### Intervention Objectives, Theory-Based Methods, Practical Strategies (Steps 2 and 3)

The goal of the second and third step was to establish the objectives of the intervention by specifying what would change as a result of the intervention. The overall aim of the intervention is to support patients in effective communication by creating awareness about the role they can play and the benefits they can gain from participating, as well as providing matching communication skills. Patient input gathered during the needs assessment was used to operationalize the overarching objective in 11 intervention objectives that relate to the awareness, attitude, and skills of the patient (Figure 3, column 1). These objectives were linked to theoretical methods and corresponding practical strategies. The main criterion for the selection of the strategies was the ability to operationalize strategies in an online environment that could be hosted by Hematon. Three theory-based methods and four practical strategies were selected to influence the attitude and skills of patients (Figure 3, columns 2 and 3).

The main method chosen was modelling. Modelling has proven to be effective in patient-targeted skill building interventions [30-33] and can be operationalized in an online environment by means of video clips. Moreover, pre-visit goal setting was selected to encourage patient involvement during the consultations. This strategy was operationalized in two ways. First, the patient’s appointment dates were linked to a reminder system, which reminds patients a week before their consultation to access the online intervention in order to prepare for their visit. Second, an open QPS was integrated, which could be completed and printed or sent to one’s personal email address. A QPS can enhance the contribution of patients in medical communication [34-36]. Finally, there was an option to store, play back, and share audio recordings of a consultation with relatives, via their personal account. Playing back audio recordings has been shown to enhance recall, improve informed decision making, reduce anxiety, and improve communication with family members [37,38].

The intervention objectives were based on the experiences expressed by the patient service users. However, because this was a more theoretical phase comprising the literature search and the analysis of data, further patient involvement in this step was limited to a discussion with the patient research partners. The outcomes were presented and the feasibility of the operationalization was discussed, which was important for the final implementation. Their feedback did not change the initial outcomes.

### Intervention Design (Step 4)

The goal of the fourth step was to design the content, structure, and layout of the intervention, inspired by the information gathered in the previous steps. An iterative design method was applied, that is, intermediate results (eg, video scripts, website navigation) were presented to patients and experts. Subsequently, the intermediate results were adapted to their feedback, which is discussed in the following paragraphs.

The targeted intervention objectives (Step 2) were translated into five video diaries, in which five simulated patients demonstrate different communication skills. Each video diary (see Multimedia Appendix 2) displays the story of one lymphoma patient in 11 to 12 short clips (47-180 seconds). This setup was chosen to capture the experiences of a large group of patients and incorporate them in five personal stories, whereas a selection of only five patients may provide a biased view [39]. The scripts for the video clips were based on personal stories that patients had expressed in Step 1. Additional material was gathered with video recordings and real-time observations of relevant hematologic consultations. This type of patient contribution represents the lowest step in the participation ladder as the involved patients agreed to be observed, but had no further understanding of the project. Subsequently, a patient research partner reviewed the scripts. The feedback contained suggestions and corresponding content for additional scenes and unclear medical/technical jargon was highlighted. We incorporated the additional scenes in the video clips and rephrased the highlighted sentences. After recording the clips, the rough material was shown to a physician, an ICT expert, a patient research partner, and an external communication researcher. Their feedback was used in the editing process. For example, the reactions of the doctors to patients’ communication behavior were cut out as a result of the feedback, aiming to increase the focus on the modelled communication behavior of the patient.

Given the changing preferences and needs of the patient, the working group chose not to present all 58 video clips to the patient at once. The patient-perceived, pre-visit communication needs determine the selection of three most relevant objectives, leading to the matching video clips. These needs are measured with an adapted version of the 10-item, 5-point Likert scaled Perceived Efficacy in Patient-Physician Interaction instrument (PEPPI). In this scale, patients indicate pre-visit their expected efficacy and post-visit their perceived momentary efficacy in communication [40]. Based on the input of patients, two extra tailored variables were added to determine which two video diaries match the patient’s situation best: (1) the patient’s preference to visit their HCP alone or with a companion, and (2) the stage of treatment (ie, ahead of treatment, in the middle of treatment, in remission, cured but monitored, and wait-and-see policy). If a patient wants to prepare his or her next consultation, new clips will be selected and these will be added to their previous selection.

Special attention was paid to two aspects that can influence the uptake of eHealth interventions: usability and credibility. According to Nielsen, usability is a quality attribute that assesses how easy user interfaces are to use and it is a necessary condition to bind users to a website. Credibility is an important element for the persuasive character of the intervention [41]. To enhance the persuasive character of the intervention, the Stanford Guidelines for Web Credibility were followed [42]. After testing preliminary versions of the intervention, a more comprehensive credibility and usability evaluation was performed by experts and prospective users. A heuristic evaluation (expert-based) and a think-aloud procedure (user-based) were set up with a total of 8 participants, which should be enough to detect over 80% of the usability problems [43]. A heuristic evaluation involves having a small set of evaluators examine the interface and judge its compliance with recognized usability and credibility principles (the heuristics). The list of heuristics used to evaluate PatientTIME was composed with the 10 usability criteria of Nielsen, supplemented with usability criteria specifically developed for older Web users [44], who are expected to be over-represented in the targeted population. The Stanford Guidelines for Web Credibility were added to this list, in order to objectively evaluate the aforementioned Web credibility. The list included themes such as consistency, user control, and efficiency. Three software experts and one master graduate in communication individually evaluated the intervention based on the list of heuristics. The user-based test included a think-aloud procedure. Two patients and two healthy people were asked to perform a set of consecutive tasks, which represented the major functionality of the intervention. Simultaneously, the subjects were encouraged to verbalize their thoughts [45]. Participants of both tests were asked to suggest improvements about the issues they came across.

The main credibility and usability issues that were identified are summarized in Table 1. Changes to these issues were incorporated before the release of PatientTIME apart from one. The illustrative pictures of patients in the layout were evaluated by the users as too positive. However, because we wanted to present a positive and encouraging context, we kept these pictures.

**Table 1:** Summary of identified credibility and usability issues.

Identified issues		Processed changes
Credibility	Information about collaborating parties, help function, and privacy issues is missing / unclear.	An extra information page was added with separate tabs containing information about collaborating stakeholders, introducing members of the working group, explaining privacy issues, and explaining the scientific context. A separate ‘help’ function was highlighted with contact details, frequently asked questions, and a project summary.
Functionality	Print function QPS unclear and use of the agenda not clear.	The agenda was made accessible on the home page, corresponding text was changed, and buttons were highlighted. The print function of the QPS^a^was highlighted.
Navigation	Location and additional text related to ‘log-in’ button is confusing. It is not always clear which elements are ‘buttons’. Not always clear where you are in the website.	The consistency in color use and type of buttons improved, more contrasting colors were used when mouse-over, headings of active pages remain highlighted and stand out more comparing to the headings of inactive pages, the home pages present instructing messages to the user about the project status.
Information	Some texts are too formal. Some inconsistency in use of terms / jargon.	Textual changes were made.
Layout	Illustrative pictures too positive / happy. Unclear presentation of the selection of video clips. It is not clear what the content of the ‘video archive’ is or will be.	Another way to present the video diaries was developed, the video archive was removed and its function was incorporated in the video page.

^a^QPS: question prompt sheet

### Development of Evaluation Plan (Step 5)

The goal of the fifth step was to develop an evaluation plan to examine the effects of the intervention. Decisions regarding the evaluation were partly stipulated in the research protocol, which proposed a randomized controlled trial (RCT) in which participants are randomized into the intervention group (with access to PatientTIME) and control group patients (without access to PatientTIME).

While working out the RCT protocol, practical issues like recruitment and patient information were discussed with the research partners and questionnaires were developed in collaboration with them. One patient research partner and one patient service user were asked to pre-test the developed questionnaires with a think-aloud procedure. Their feedback focused mainly on questions initially formulated as too formal or medical jargon that was unclear.

The involvement of prospective participants (ie, patient service users) in the RCT was planned on different levels. Both intervention and control group participants were asked to participate for a maximum of three consultations and they were both asked to fill in questionnaires delivered via their personal account. On the lowest participation level, participants are provided with information and asked to complete questionnaires. On a second level, they are encouraged to verbalize their ideas and input with regard to the study design to inform decisions taken by the working group. Last, a random subset of patients in the intervention group is encouraged to audio record and upload their consultation(s) on their secured PatientTIME account. This pilot was designed for the purpose of evaluating the playback option as well as to be analyzed by the researchers on their actual participation during their consultation.

The developed RCT protocol was audited with external experts to evaluate privacy issues and the exchange of online information and to assess and reduce possible risks. Because of the juridical, technical nature of the audit, we did not include patients in this audit. The Medical Ethical Committee of the Radboud University Nijmegen Medical Centre evaluated the RCT protocol and concluded that following the Dutch Medical Research Involving Human Subjects Act, the study did not require ethics approval. The RCT (registered in the Netherlands Trial Register, 3779) started in 2013 and the first results are expected to be available in 2015.

### Development of Implementation Plan (Step 6)

The goal of the last step was to design an implementation plan that would guide the transfer of the intervention to a publicly available online tool. Contrary to the detailed evaluation plan, the implementation plan was a rough setup of actions that were guided by and adapted to decisions made in previous steps. To increase the chance of a successful implementation and adoption, the involvement of patients and Hematon in the planning and execution of the actual implementation started as early as the project planning. In the preparatory step, the board of Hematon was asked to help thinking about the valorization of the research results. In this way, we aimed to divide responsibilities at an early stage and awareness was created about the upcoming intervention.

Hematon wanted to make developed materials available for all their members and other patients. As a result, an agreement was established noting that after research is finalized, Hematon would become responsible for hosting the tool. Subsequently, during the development of the intervention and evaluation plan, several meetings were planned with our software developer and the webmasters of Hematon. In consultation with them, we aimed to develop materials that were not only usable for the secured trial setting, but could easily be transferred to a publicly available tool. Both patient research partners will be actively involved in the actual transfer of the intervention.

This transfer is not within the scope of this paper and will be done when the RCT proves to be acceptable, usable, and efficient. Lessons learned from the evaluation will be used to optimize the intervention before implementation.

## Discussion

### Principal Findings

In the PatientTIME project, patients were given the opportunity to actively participate in the development of an online communication intervention with corresponding evaluation and implementation plan. In conformity with previously publications, the cooperation with patients brought valuable insights and appeared to influence many decisions made [46,47]. By combining patient participatory methods with a theoretical protocol, we aimed to create a bottom-up inspired development procedure. We encountered both facilitating elements, as well as obstacles in this approach.

### Facilitators to Participatory Development

The combination of evidence-based and patient participatory methods did assist us in involving patients. The structure of the IM framework helped us choose when to involve patients, while the idea of participation ladders and user-centered design thinking inspired us in how to involve patients.

The involvement of patients on different levels appeared to be useful and practical. The patient research partners ensured a continuous patient-centered view, while the patient service users were able to give fresh new insights on different protocol steps.

Both Hematon as well as the research partners were involved from the very beginning of the project as a result of the preparatory planning step. We experienced this as a precondition to creating a continuous patient-centered view. Their early involvement supported the participation of patient service users and it gave the opportunity to discuss possible valorization of results at an early stage.

Another facilitating aspect was the attitude of the participating patients. They all seemed to recognize why the intervention was developed. This appeared to be a driving force behind their motivation to participate. Attracting engaged patients may be a precondition to creating a successful patient-centered approach.

### Obstacles to Participatory Development

The recruitment and involvement of patients was a time-consuming part of the project. In some steps, we could have benefited from more involved patient service users (especially the intervention development step), but time constraints prevented us from doing so. The extent of patient involvement relates to the amount of time available to execute the project. However, we think time constraints should not be a reason for limited participation.

Flexibility in terms of planning and setup seemed a precondition to including the perspectives of the (seriously ill) patients. For example, during the needs assessment, some patients were too ill to attend a focus group session. An interview at their home gave us the opportunity to incorporate their experiences as well. Considering the illness of the targeted patients, we think the extent of involvement of service users should be evaluated per protocol step.

Flexibility also appeared to be a key concept in incorporating patients’ viewpoints and experiences in the defined research proposal. In the current study, a research proposal defined certain decisions, for example, the intervention would be delivered online and the evaluation of the effects would be tested in an RCT. Although the proposal was built on previous research and experiences, these decisions were made before the targeted patients could be consulted (see Future Research).

### Future Research

While there is a desire for more patient participation in research, it seems to clash with strict research proposals and protocols that need to be approved before the start of a project. Perhaps researchers should involve (ex-) patients in the design of such documents. However, this still does not give the required flexibility to adapt a project to the input of patients, gathered along the way. Patient participation in research projects that include design activities requires methodologies that allow the dynamics of design (eg, by patient input) to influence the process. Intervention mapping can be a guiding method, unless it is bounded to a strict predefined proposal. Participatory Learning and Action Research or Design Inclusive Research might be interesting alternative methodologies [48-50]. Funders also should evaluate the extent of detail they request in proposed projects and how this might restrict the extent of (true) influence patients can have.

Considering the evaluation of online interventions and the necessary flexibility to incorporate patients’ input, it might be interesting to study other perhaps more flexible evaluation methods than an RCT. A longitudinal study where intermediate results can be used to optimize the intervention during the test phase might be an interesting alternative. Furthermore, some patients might have a strong preference for using or not using technology. In the case of strong preferences, results may be biased when using a regular randomized controlled trial. Within preference trial designs, this bias is dealt with by the fact that patients with strong preferences for either intervention will get the intervention they prefer. Only those without explicit preference are randomly assigned to either the intervention or the control group [51].

### Limitations

A limitation of the applied method is that the participating patients represent a self-selected convenience sample as involved patients voluntary signed up to contribute to the study. This could have led to a biased view of a more empowered group of patients. In general, the possibility of having a biased group of participants in a participatory development approach is evident, as one needs to find patients that are interested in cooperating. On the other hand, one wants to develop an intervention that reaches out to the whole targeted population. This advocates the use of different participation levels and creative solutions to attract and/or select patient service users to capture a broad view of experiences.

Similar to other studies [52], in the current study the IM framework was not applied in a linear way as proposed, which can be argued as a potential limitation. However, a design process rarely follows a parallel execution process and, especially because the aforementioned flexibility was required, we think it does not have to affect the quality of the developed products.

### Conclusions

Involvement of patient research partners in combination with patient service users can inspire and guide the evidence-based intervention mapping protocol. Early involvement, involvement on different levels, and flexibility in terms of planning and setup seem to be preconditions to create a bottom-up inspired development procedure with (seriously ill) patients. Further research is necessary to find out if a more patient-centered approach improves the implementation and uptake of eHealth interventions.

## Multimedia Appendix 1

Intervention.

## Multimedia Appendix 2

Video diaries.

## Abbreviations

HCP: health care professional
ICT: information and communication technology
IM: intervention mapping framework
PatientTIME: Patients Talk in Medical Encounters
PEPPI: Perceived Efficacy in Patient-Physician Interaction
QPS: question prompt sheet
RCT: randomized controlled trial
UCD: user-centered design
