# Supplementary material for: Active Patient Participation in the Development of an Online Intervention
Source: JMIR Res Protoc. 2014 Nov 6;3(4):e59. doi: 10.2196/resprot.3695 (PMC4259996; doi:10.2196/resprot.3695)
Supplement: Supplementary file 1 [file resprot_v3i4e59_app1.pdf]

Online communicatietraining
voor (non-)Hodgkinlymfoom patiënten

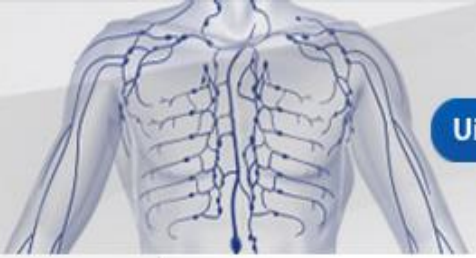

Uitloggen

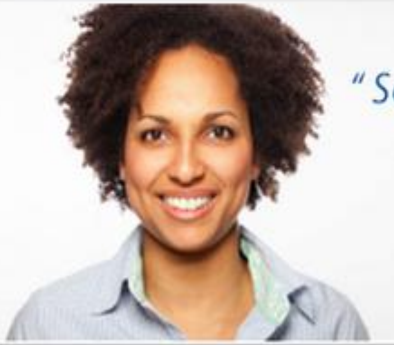

" Soms klap je gewoon dicht..."

Welkom
Inge van Bruinessen

Home | Vragenlijst | Mijn video's | Actielijst | Help

Mijn agenda

Home

Beste Inge van Bruinessen,

Welkom op uw persoonlijke pagina van de PatientTIME website.
Uw programma start één week voor uw volgende afspraak.

De datum van uw volgende afspraak is ingevuld in 'Mijn Agenda' (zie rechter kolom)
Klopt de datum niet of staat er nog geen datum ingevuld?
U kunt de datum aanpassen door erop te klikken.

Met vriendelijke groet,
Het PatientTIME team

Volgende afspraak is op:

geef een datum ...

opslaan

Vorige afspraken:

06 juni 2013
