# Supplementary material for: Active Patient Participation in the Development of an Online Intervention
Source: JMIR Res Protoc. 2014 Nov 6;3(4):e59. doi: 10.2196/resprot.3695 (PMC4259996; doi:10.2196/resprot.3695)
Supplement: Supplementary file 2 [file resprot_v3i4e59_app2.pdf]

Video dagboek Emma

< terug naar overzicht dagboeken

- Maak kennis met Emma
- Terugblik van Emma
- Voor een consult
- Emoties tijdens een consult
- Vragen stellen
- Alle vragen gesteld krijgen
- Complexe informatie
- Informatie delen met de arts
- Behoefte aan ondersteuning
- Verschillende informatiebronnen
- Onvrede

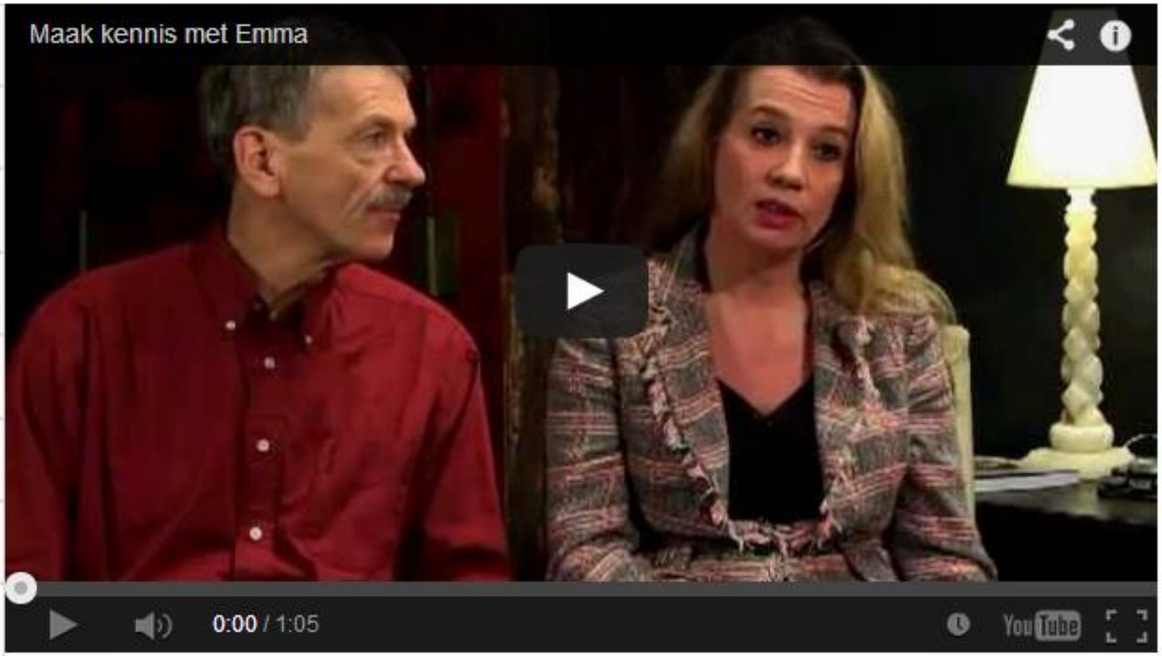

type hier uw notities voor uw actielijst

Voeg toe aan actielijst
